# Cytosolic and Calcium-Independent Phospholipases A_2_ Activation and Prostaglandins E_2_ Are Associated with *Escherichia coli*-Induced Reduction of Insulin Secretion in INS-1E Cells

**DOI:** 10.1371/journal.pone.0159874

**Published:** 2016-09-15

**Authors:** Nunzia Caporarello, Mario Salmeri, Marina Scalia, Carla Motta, Cristina Parrino, Lucia Frittitta, Melania Olivieri, Martina Cristaldi, Roberto Avola, Vincenzo Bramanti, Maria Antonietta Toscano, Carmelina Daniela Anfuso, Gabriella Lupo

**Affiliations:** 1 Dept. of Biomedical and Biotechnological Sciences, School of Medicine, University of Catania, Catania, Italy; 2 Dept. of Clinical and Experimental Medicine, School of Medicine, University of Catania, Catania, Italy; Universidade Federal do Rio de Janeiro, BRAZIL

## Abstract

It is suspected that microbial infections take part in the pathogenesis of diabetes mellitus type 1 (T1DM). Glucose-induced insulin secretion is accompanied by the release of free arachidonic acid (AA) mainly by cytosolic- and calcium independent phospholipases A_2_ (cPLA_2_ and iPLA_2_). Insulinoma cell line (INS-1E) was infected with *E*. *coli* isolated from the blood culture of a patient with sepsis. Invasion assay, Scanning Electron Microscopy and Transmission Electron Microscopy demonstrated the capacity of *E*. *coli* to enter cells, which was reduced by PLA_2_ inhibitors. Glucose-induced insulin secretion was significantly increased after acute infection (8h) but significantly decreased after chronic infection (72h). PLA_2_ activities, cPLA_2_, iPLA_2_, phospho-cPLA_2_, and COX-2 expressions were increased after acute and, even more, after chronic *E*. *coli* infection. The silencing of the two isoforms of PLA_2_s, with specific cPLA_2_- or iPLA_2_-*si*RNAs, reduced insulin secretion after acute infection and determined a rise in insulin release after chronic infection. Prostaglandins E_2_ (PGE_2_) production was significantly elevated in INS-1E after long-term *E*. *coli* infection and the restored insulin secretion in presence of L798106, a specific EP3 antagonist, and NS-398, a COX-2 inhibitor, and the reduction of insulin secretion in presence of sulprostone, a specific EP3 agonist, revealed their involvement in the effects triggered by bacterial infection. The results obtained demonstrated that cPLA_2_ and iPLA_2_ play a key role in insulin secretion process after *E*. *coli* infection. The high concentration of AA released is transformed into PGE_2_, which could be responsible for the reduced insulin secretion.

## Introduction

Research in recent years has turned its attention to the bacterial infections that develop in patients with diabetes [1, 2]. But could it be that a generalized bacterial infection is able to reduce the secretion of insulin by pancreatic cells and consequently have a causal role in diabetes? Microbes, viruses in particular, have been the focal point of diabetes research for several decades but proving a causal role between infection and the onset of diabetes mellitus type 1 (T1DM) is, however, extremely difficult. One of the reasons is the long period between exposure and the clinical onset of the disease. Another problem is that affected individuals often experience multiple infections over the years before the onset of T1DM, as do non-diabetics in the population [3].

Several mechanisms have been proposed for explaining how bacteria are able to damage pancreatic cells. Streptomyces strains may act by producing a toxin that would affect the pancreatic ß cells causing their lysis [4]. In other cases the bacterial infection would result in the activation of lymphocytes and an increase in the concentration of cytokines in close proximity of the pancreatic cells [5, 6]. It has been demonstrated that endotoxins, released during bacterial infection, induced apoptosis in insulin secreting (INS-1) cells [7], caused acute insulin resistance, followed by long-lasting tissue-specific dysfunctions of lipid and glucose metabolism [8] and could deteriorate insulin secretion in a rodent model of metabolic syndrome [9].

In addition, hyperglycemia, associated with hypoinsulinemia, may be the normal pathophysiological response in children with meningococcal sepsis [10] suffering from frequent and significant hyperglycemic episodes associated with low insulin levels in the plasma during the acute phase of the disease [11]. The results of a study of obese and non-obese dogs show that *Staphylococcus intermedius* infection is able to reduce insulin sensitivity in mongrel dogs [12]. *Salmonella typhimurium* has been identified as a causative agent of acute pancreatitis [13]; *Salmonella* persistent infection is characterized by a loss of pancreatic acinar cells and accumulation of inflammatory cells, being able to colonize the pancreas *in vivo*, and to invade cultured pancreatic acinar cells *in vitro* [14]. Moreover, acute pancreatitis is a recognized complication of Hemolytic Uremic Syndrome in the setting of *E*. *coli* infection [15]. There may be a percentage of patients with *E*. *coli* colitis with undiagnosed pancreatitis [16]. It has been demonstrated, in a cat model, that bacterial infection is able to trigger acute pancreatitis [17]. In rabbit, acute pancreatitis can be induced by infected bile, which causes an interstitial-edematous trait with occasional acinar necrosis, its severity depending on the bacterial species, including *E*. *coli* [18].

*E*. *coli* normally colonizes the gastrointestinal tract in infants a few hours after birth. These commensal strains of *E*. *coli* rarely cause disease except in immuno-compromised patients [19] or where the normal gastrointestinal barriers have been altered as in the case of peritonitis [20]. However, there are several *E*. *coli* strains which acquire specific virulent characteristics, becoming capable of adapting to new niches. These attributes of virulence are often encoded on genetic elements that make some *E*. *coli* strains capable of causing diseases in healthy individuals [21].

Most of the pathogenic *E*. *coli* strains remain extracellular, but enteroinvasive *E*. *coli* (EIEC) is a true intracellular pathogen that is capable of invading and replicating within epithelial cells and macrophages [22].

The early phase of EIEC pathogenesis comprises epithelial cell penetration, followed by lysis of the endocytic vacuole, intracellular multiplication, directional movement through the cytoplasm and extension into adjacent epithelial cells [23]. Movement within the cytoplasm is mediated by nucleation of cellular actin into a ‘tail’ that extends from one pole of the bacterium [24]. Through this pathogenic mechanism, *E*. *coli* could infect different organs including the pancreas, leading to a reduction of insulin secretion. On the other hand, it is right to report that *in vitro* studies have shown that the presence of bacteria can reduce or even increase insulin secretion in cultures of pancreatic tissue, depending on the type of infecting microorganism. The infection by *Pseudomonas* causes reduction of insulin secretion while *Enterobacter* and *Staphylococcus* determined an increase in insulin secretion [25]. These conflicting results require further studies that may elucidate the molecular mechanism that induces the onset of diabetes in patients with bacterial infection.

Arachidonic acid (AA) is released from membrane phospholipids by the action of the different isoforms of phospholipases A_2_ (PLA_2_) and converted into prostaglandin (PGs) or leukotrienes (LTs) by the action of cyclooxygenases (COX-1 and COX-2) and 5-lipoxygenase, respectively. Cytosolic PLA_2_ (cPLA_2_), Ca^2+^-independent PLA_2_ (iPLA_2_), and Ca^2+^-dependent secretory PLA_2_ (sPLA_2_) differ from each other in terms of substrate specificity, Ca^2+^-requirement, modification of lipids, translocation to cell membranes, and the release of AA [26].

The cPLA_2_, present in many cell types, including pancreatic ß cells (cPLA_2_ß), requires phosphorylation at Ser-505 and binding with Ca^2+^ for its activity [27]. cPLA_2_ß stimulates insulin exocytosis by accelerating granule mobilization and “overfilling” of the readily releasable pool so that more granules are available for release once intracellular Ca^2+^ concentration rises to exocytotic levels. Activation of cPLA_2_ß during the ß-cell stimulus/secretion coupling would cause translocation of the enzyme to the secretory granules and accumulation of AA and lysophospholipids in the membrane, leading to changes in membrane structure or fluidity [27]. It has also been demonstrated that cPLA_2_ß plays a role in the maintenance of insulin stores, but it is not required for the initiation of insulin secretion from ß-cells [28]. Moreover, overexpression of cPLA_2_ results in severe impairment of the calcium and secretory responses of ß-cells to glucose through upregulation of mithocondrial uncoupling protein-2 [29]. In pancreatic ß cells, the enzyme iPLA_2_ß does not require Ca^2+^ for the catalytic activity and it is inhibited by the suicide substrate bromoenol lactone (BEL) [30]. It has been shown that iPLA_2_ß is involved in apoptosis of ß-cells of the pancreas during diabetes and its inhibition is able to reduce apoptosis, thus preventing cell dysfunction associated with diabetes [31]. Type IB sPLA_2_ is contained in insulin secretory granules of pancreatic islet ß-cells, it is co-secreted with insulin from glucose-stimulated islets [32] and it is expressed in human islets of transplanted pancreas after the recurrence of type 1 diabetes mellitus with insulitis [33].

The AA, produced by PLA_2_ activities, has a significant regulatory action on insulin secretion in pancreatic ß cells [34].

Our previous studies showed the significant role of cPLA_2_, iPLA_2_ and PKCα/ERK/MAPK signalling pathways during *E*. *coli* infection of microvascular endothelial cells [35, 36]. Moreover, we demonstrated that *S*. *aureus* chronic infection of INS-1 cells causes a decrease in insulin release and a significant increase of cPLA_2_, iPLA_2_ activity/expression and COX-2 protein expression [37].

The objective of this study was to investigate the role of the PLA_2_s in the *E*. *coli* infection of ß cells and the molecular mechanisms which could lead to T1DM pathogenesis.

To carry out this study, we used *E*. *coli* isolated from the blood of a female patient dying from severe sepsis with underlying acute pyelonephritis and subsequent multiple-organ failure. To gain insight in the correlation between bacterial infection-response of the pancreas in terms of insulin secretion, we performed *E*. *coli*-infection experiments by using the insulin-producing INS-1E rat cell line, which is widely used as a pancreatic ß-cell model, retaining glucose-stimulated insulin secretion and a high degree of differentiation [38]. Here we demonstrated that *E*. *coli* is able to enter INS-1E cells in a time-dependent manner. Chronic infection causes a significant decrease in insulin release and a significant PLA_2_s and COX-2 protein activation. Furthermore, we provide data suggesting that prostaglandin E2 (PGE_2_) production plays a key role in the reduction of insulin secretion after long-term infection and that insulin secretion by *E*. *coli*-infected ß-cells could be restored by using specific *si*RNAs against cPLA_2_ and iPLA_2_ isoforms.

## Material and Methods

All reagents and antibodies were purchased from Sigma or E. Merck unless otherwise indicated. Phospholipase A_2_ inhibitors, arachidonoyl trifluoromethyl ketone (AACOCF3), and bromoenol lactone (BEL) were from Calbiochem. Phospholipase A_2_ (cytosolic and calcium independent), sPLA_2_ specific activity assay kits, rabbit polyclonal against iPLA_2_ antibody and Sulprostone were from Cayman Chemical (Ann Arbor, MI). cPLA_2_ (*mouse monoclonal*), phospho-cPLA_2_ (*rabbit polyclonal*), anti-α-actin (*mouse monoclonal*), anti-COX-1 and anti-COX-2 (*mouse monoclonal*) were purchased from Santa Cruz Biotechnology.

### Bacterial strains and culture conditions

*E*. *coli* strain belongs of a collection of a hospital laboratory of Clinical Microbiology and has been isolated from blood culture of a patient with sepsis. All patient data have followed the required anonymity procedure, being the patient identified with alphanumeric code. As the strain is of collection, the Ethics Committee did not have to be approached.

*E*. *coli* was grown in tryptic soy broth (TSB) at 37°C for 14h. The culture was centrifuged at 4300xg for 10 min, and the supernatant discarded. The bacterial pellet was washed with PBS and serially diluted to the desired concentration. The density of bacteria was measured by enumerating the number of CFU on LB agar plates (Difco).

### Cell cultures

Rat insulinoma β-cell line (INS-1E) was kindly provided by Dr. C. B. Wollheim, (Médical Universitaire, Genève, Switzerland). Cells were cultured in RPMI-1640 medium containing 5 mM glucose, supplemented with 10% heat-inactivated fetal bovine serum, 100 U/mL penicillin, 100 U/mL streptomycin, 1 mM sodium pyruvate and 50 μm β-mercaptoethanol in 5% CO_2_ atmosphere at 37°C [39].

### Invasion assay

Cell monolayers (grown in 6-well tissue culture plate at a density of 8 x 10^5^ cells/well) were infected with *E*. *coli* (10^7^ CFU/well) for 2h, 4h, 6h and 8h in a serum free medium. At the end of the incubation times, gentamicin at 100 μg/ml (Sigma-Aldrich) was added and left for 1 h to kill extracellular bacteria. Cells were then washed three times with PBS, lysed with 1 ml of 0.1% Triton X-100 in PBS, and plated onto LB agar plates with the appropriate antibiotics. Invasion frequencies were calculated as the number of bacteria surviving the incubation with gentamicin divided by the total number of bacteria present in the absence of the antibiotic. The experiments were performed three times in triplicate on separate days, and the data was expressed as percentage of invasion.

### Cell viability

In order to determine the viability of INS-1E cells after short-term and long-term *E*. *coli* treatment, cells were trypsinized, cell suspensions were mixed with a 0.4% (w/v) trypan blue solution, and the number of live cells was determined using a haemocytometer. Cells failing to exclude the dye were considered non-viable. Each infection was performed in triplicate and counted four times each.

### Electron microscopy

For Scanning Electron Microscopy (SEM) preparations, INS-1E cells, grown on sterile circular cover glasses, inserted into 6-well chamber slide (8 x 10^5^ cells/well), and infected for 8h with *E*. *coli* (10^7^ CFU/well) were fixed with 1.5% glutaraldehyde in 0.12 M phosphate buffer (pH 7.5) overnight at 4°C. Cells were then postfixed in 1% OsO_4_ for 1 h at 4°C. Following washing with distilled water, the cells were dehydrated in graded ethanol, critical point dried, and sputtered with a 5-nm gold layer using an EmscopeSM300 (Emscope Laboratories, Ashford, United Kingdom). They were then observed using a Hitachi S-4000 (Hitachi High-Technologies America, Inc., Schaumburg, IL) field emission scanning electron microscope. For transmission electron microscopy (TEM), after being dehydrated in a graded series of acetone, cells were embedded in Durcupan ACM (Fluka Chemika-Biochemika, Buchs, Switzerland). Ultrathin sections were cut perpendicularly from the membrane using a Reichert Ultracut E microtome and double stained with uranyl acetate and lead citrate. Observations were carried out using a Hitachi H-7000 transmission electron microscope (Hitachi High-Technologies Europe GmbH, Krefeld, Germany).

### Insulin secretion assay

Glucose-induced insulin secretion was evaluated as previously described [39]. INS-1E cells (8 x 10^5^ cells/well) were seeded in 6-well plates and incubated for 8h in a serum free medium containing *E*. *coli* (10^7^ CFU/well). At the end of the incubation period, the medium containing bacteria was removed and gentamicin at 100 μg/ml (Sigma-Aldrich) was added and left for 1h to kill extracellular bacteria. Cells were then washed three times with PBS and cultures were randomly divided into two groups to mimic an acute and a chronic infection. The first group (short-term infection) was stopped at this point (after incubation with *E*. *coli* for 8h) miming an acute infection. The second group (long-term infection), after 8h of infection with *E*. *coli*, was further incubated for another 72h in a bacteria-free medium containing 5 mM glucose in order to allow bacterial proliferation inside the cells and to mimic a chronic infection. Cells from the two groups were then incubated for 1h at 37°C in Krebs-Ringer-HEPES buffer (KRHB) [39] containing 2.7 mM glucose (starvation). Thereafter, cells were washed with KRHB and incubated for 2h in the same buffer containing different concentrations of glucose (5.5 mM, 11.1 mM, 16.6 mM and 22.2 mM). Aliquots of supernatant were taken for the measurement of insulin secretion, while total protein content was determined by using BCA protein assay (Pierce). Non-infected cells (control cells) were incubated in a bacteria-free medium for the same incubation time as infected cells, for 8h (control cells of short-term *E*. *coli* infection) and for 8h plus 72h (control cells of long-term *E*. *coli* infection). In the experiments in presence of inhibitors, INS-1E cells were pre-incubated for 60 min in culture medium supplemented or not with 5 μM NS-398, COX-2 specific inhibitor, or 20 μM L-798106, specific EP3 antagonist, or 10 nM sulprostone, specific EP3 agonist.

The cells were then re-fed with fresh culture medium containing the inhibitors in presence or in absence of *E*. *coli* for 8h (short-term infection) or for 8h and subsequently further incubated for 72h (long-term infection).

Insulin levels in the culture media were measured by ELISA kit (Millipore). Data were expressed as percentage of maximal secretion showed in INS-1E cells, which for glucose stimulation was obtained at 16.6 mM.

### Phospholipases A_2_ activity assay

INS-1E cells were pre-incubated for 1h in RPMI 1640 medium containing 5 mM glucose, supplemented or not with either 50 μM AACOCF3 (Arachidonoyl trifluoromethyl ketone, both PLA_2_s activity blocker) or 2.5 μM BEL (Bromoenol lactone, specific iPLA_2_ inhibitor) or 5 mM EDTA (cPLA_2_ inhibitor). The cells were then re-fed with fresh culture medium containing the inhibitors, in the presence or in the absence of *E*. *coli* (10^7^ CFU/well) for 8h. At the end of the incubation period, cells were divided into two groups and processed as described in order to mime an acute and a chronic infection. Cultures from short-term infection (cells incubated for 8h with *E*. *coli* in the presence or absence of inhibitors) and from long-term infection (cells incubated for 8h with *E*. *coli* in the presence or absence of inhibitors and subsequently further incubated for 72h in the presence or absence of inhibitors) were lysed as previously described [40], and lysates were used for cPLA_2_ and sPLA_2_ activity assays, following the manufacturer’s instructions. Results were expressed as a percentage compared to the control non-infected cells.

### Immunoblotting

For immunoblotting, INS-1E cells from short and long-term *E*. *coli* infection were collected by trypsinization. Controls were performed with non-infected cells. The lysates of INS-1E cells were prepared for Western blotting as previously described [41, 42]. The protein content of the cell lysate was quantified by BCA assay. Membranes were incubated with primary antibodies (1:500 dilution) against total cPLA_2_, iPLA_2_, phospho-cPLA_2_, COX-1, COX-2 and α-actin, and then incubated with secondary antibodies for 1h at room temperature.

### Transfection of siRNAs

The cPLA_2_ and iPLA_2_ knock-down in INS-1E cells was carried out by using rat ON-TARGET plus SMART pool *si*RNA duplex (Dharmacon, Chicago, IL), transfected by Lipofectamine RNAiMax (Life Technologies, CA, USA). Two sets of oligonucleotides were used: the first direct against cPLA_2_ (Gene Bank NM_133551) and the second one direct against iPLA_2_ (Gene Bank NM_001005560). *si*RNA used were provided as SMART pool designed against shared and conserved regions in order to ensure efficient and specific target silencing for cPLA_2_ α, β and γ, as well as iPLA_2_, as indicated by the provider. A *si*RNA non targeting was used as negative control, according to the manufacturer’s instruction. Western blot analysis confirmed the reduction of the protein target level. After transfection with iPLA_2_-*si*RNA or cPLA_2_-*si*RNA, the cells were infected for 8h with *E*. *coli* (10^7^ CFU/well). At the end of the incubation period, the cells were divided in two groups, as described (long- and short-term infection), and insulin release was determined.

### Determination of PGE_2_ production

To determine PGE_2_ production, INS-1E were pre-incubated for 60 min in culture medium supplemented or not with either 50 mM AACOCF3 or 2.5 mM BEL. The cells were then re-fed with fresh culture medium containing the inhibitors in presence or in absence of *E*. *coli* for 8h (short-term infection) or for 8h and subsequently further incubated for 72h (long-term infection). Supernatants were collected and aliquots were employed for PGE_2_ determination, by kit from Cayman Chemicals, Ann Arbor, MI, USA. For PGE_2_, the detection range was 7.8–1000 pg ml^-1^.

### Statistical analysis

Data is reported as mean ± standard deviation (SD). Statistical significance between two groups was analyzed by Student’s *t-*test. One-way analysis of variance (ANOVA), followed by Tukey’s *post-hoc* test, was used to compare the means for the multiple groups. GraphPad Prism was used to generate bar graphs. The *p* value <0.05 was considered statistically significant.

## Results

### Capability of *E*. *coli* to enter INS-1E

**Fig 1:**
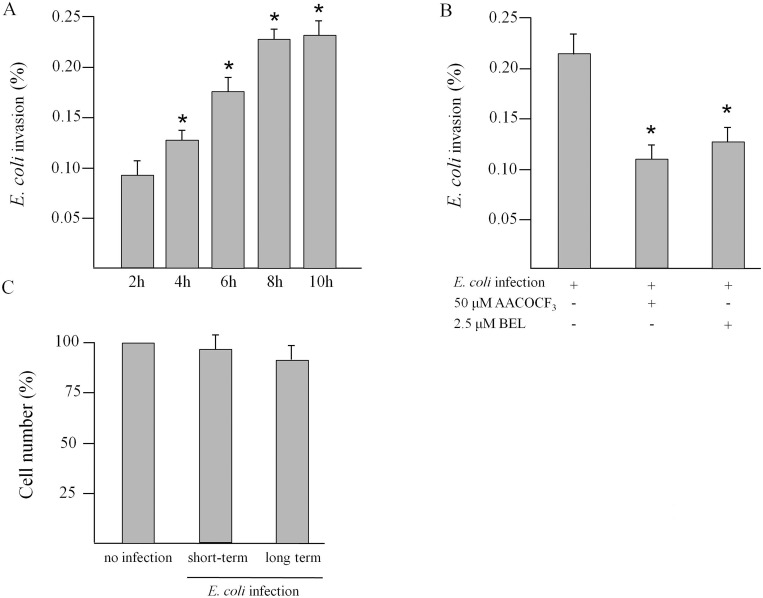
Invasion assay, in absence or in presence of PLA_2_ inhibitors, and viability after incubation of INS-1E cells with *E*. *coli*. Panel A: invasion of INS-1E cells with *E*. *coli* for 2h, 4h, 6h, 8h and 10h. Values are expressed as a percentage of invasion ± SD by three independent experiments performed in triplicate. Statistically significant differences, by one-way ANOVA and the Tukey *post-test* are indicated (*p<0.05 *vs* 2h invasion). Panel B: effect of PLA_2_ inhibitors (50 μM AACOCF3 or 2.5 μM BEL) on 8h *E*. *coli* invasion of INS-1E cells. Values are expressed as a percentage of invasion ± SD by three independent experiments performed in triplicate. Statistically significant differences, by one-way ANOVA and the Tukey *post-test* are indicated (*p<0.05 *vs* 8h invasion without inhibitors). Panel C: number of live non-infected cells and after short-term and long-term infection (see Materials and Methods). Values, in percentage compared to control cells incubated in absence of bacteria (mean ± SD) are from three independent experiments (n = 3).

**Fig 2:**
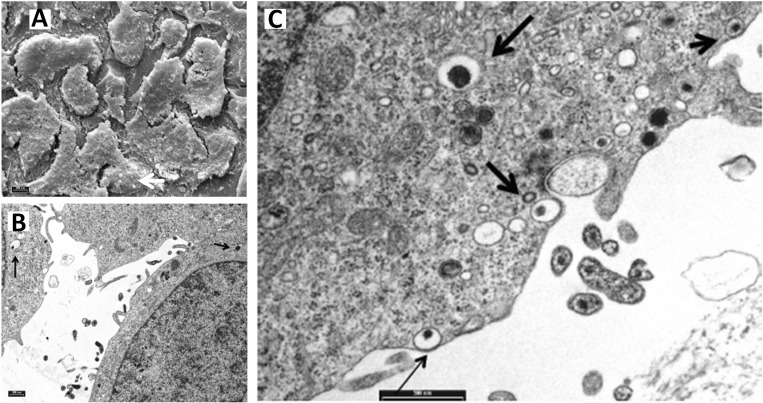
Scanning Electron Microscopy (SEM) and Transmission Electron Microscopy (TEM) of INS-1E cells infected with *E*. *coli* for 8h. (A) The surface of the cells shows microvilli variable in size and in shape and some bacteria on cell surface (white arrow) are visible (magnification, x 2000). (B) Numerous bacteria are present between the cells, in contact with pseudopod-like structures on the surface of the cells and some bacteria are engulfed in the cytoplasm of the cells (arrows). Bar 500 nm. (C) The bacteria appear to be in close contact with the cell membranes which encircle the microorganism (arrows). Some bacteria are engulfed intracellularly inside membrane-bound vacuoles (arrow). Bar 500 nm.

To evaluate the capability of *E*. *coli* to enter INS-1E cells, invasion assays were performed (Fig 1A). The percentage of invasion at 4h, 6h and 8h increased 1.6-, 2.2 and 2.9 fold respectively in comparison with invasion after 2h. The number of invasive bacteria recovered after 10h was very similar to that of 8h, indicating that the greatest number of bacteria was able to enter cells after 8h of incubation. For this reason, 8h incubation time was chosen for all the infection experiments. As shown in Fig 2B, the infection with *E*. *coli* for 8h of INS-1E in presence of PLA_2_ activity dual blocker AACOCF3 or iPLA_2_ inhibitor BEL caused a significant inhibition of invasion by about 50% and 40%, respectively, compared to invasion in absence of inhibitors. Trypan blue exclusion test demonstrated that short-term and long-term infection with *E*. *coli* (see Materials and Methods) did not affected cell viability (panel C).

Fig 2A shows Scanning Electron Microscopy (SEM) images of INS-1E cells after 8h infection with *E*. *coli*. Some bacteria on the surface of the cells are visible (white arrow). Fig 2B and 2C show Transmission Electron Microscopy (TEM) images of INS-1E cells after 8h infection with bacteria. Pseudopod-like structures indicate that invasion requires cytoskeletal rearrangements. Some bacteria are present in the cytoplasm of the cells within vacuoles (black arrows).

### Insulin secretion after acute and chronic *E*. *coli* infection

**Fig 3:**
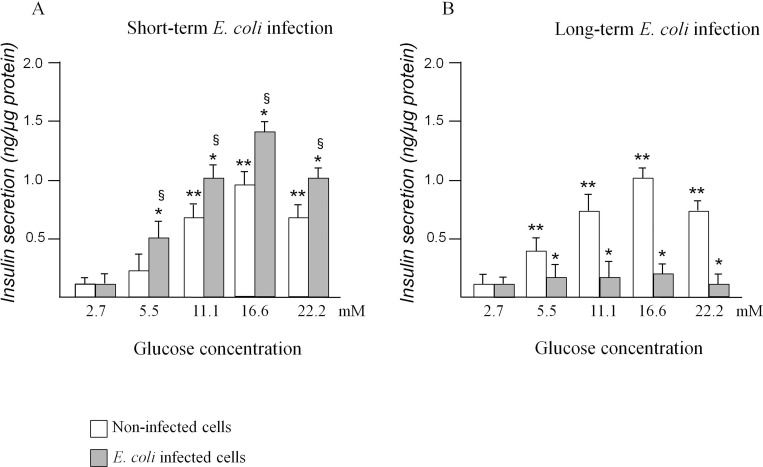
Insulin release in INS-1E cells infected for short-term (8h, panel A) or long-term (8h plus 72h, B) with E. coli. Control cells were incubated in a medium without bacteria for 8h (in short-term *E*. *coli* infection experiments) or for 8h plus 72h (in long-term infection experiments). Values are expressed as ng/μg protein (mean ± SD measured by three independent experiments performed in triplicate). Statistically significant differences, by one-way ANOVA and the Tukey *post-test* (p< 0.05) are indicated: (*) infected *vs* non-infected cells at the same glucose concentrations; (**) non-infected at different glucose concentrations *vs* non-infected cells at 2.7 mM glucose concentrations; (§) infected at different glucose concentrations *vs* infected cells at 2.7 mM glucose concentration.

Insulin release was determined in INS-1E cells after short-term (8h, acute) infection (Fig 3A) and after long-term (72h, chronic) infection (Fig 3B). In non-infected cells (control), the insulin release in presence of 16.6 mM glucose (maximal secretion) was 0.94 ± 0.05 ng/μg protein/h. The insulin release after short-term infection significantly increased 1.5 fold in presence of 16.6 mM glucose concentration in comparison to non-infected cells (panel A). After long-term infection, insulin release in presence of 16.6 mM glucose concentration significantly decreased 5.5 fold in comparison to non-infected cells at the same glucose concentration (panel B).

### PLA_2_ activities

**Fig 4:**
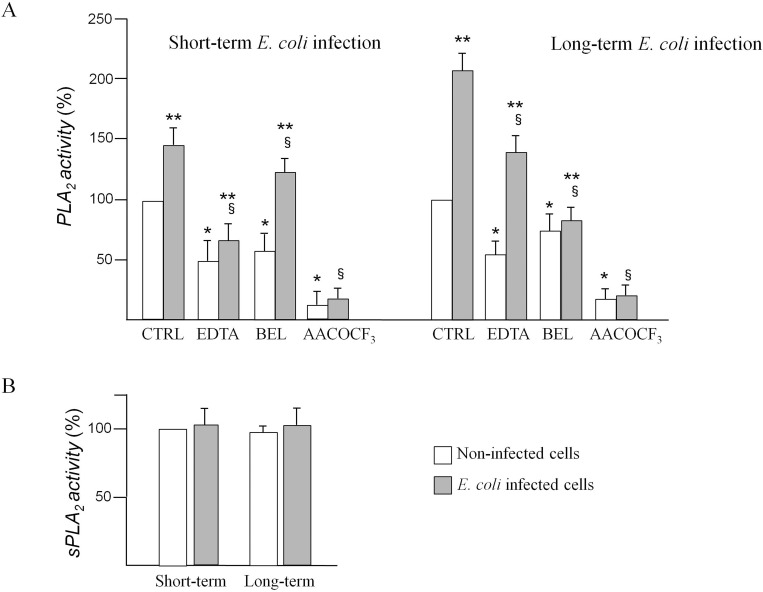
Phospholipase A_2_ activities in INS-1E cells. cPLA_2_ and iPLA_2_ activities in non-infected cells and after short-term infection (panel A) or after long-term infection (panel B) in absence or in presence of 50 μM AACOCF3 or 2.5 μM BEL or 5 mM EDTA (see Materials and Methods). Panel C: sPLA_2_ activity in non-infected and in infected cells (short-term and long-term infection) with *E*. *coli*. Values, in percentage compared to control cells incubated in absence of bacteria (mean ± SD) are from three independent experiments (n = 3). Statistically significant differences, by one-way ANOVA and the Tukey *post-test* (p< 0.05) are indicated: (*) non-infected cells with inhibitors *vs* non-infected w/o inhibitor cells; (§) infected cells with inhibitors *vs* infected cells w/o inhibitors; (**) infected *vs* non-infected in absence or in presence of the same inhibitor.

Fig 4 shows PLA_2_ activities in INS-1E cells non-infected or infected (short- and long-term infection) with *E*. *coli* in absence or in presence of PLA_2_ inhibitors. The use of EDTA, and BEL in control and infected cells allowed us to discriminate between cPLA_2_ and iPLA_2_ activity. The enzyme activity insensitive to BEL represents Ca^2+^-dependent PLA_2_ contribution, whereas the enzyme insensitive to EDTA represents Ca^2+^-independent PLA_2_. None of these components, used at the specified concentration, affected cell viability, as verified by trypan blue exclusion test (data not shown). Results, pmol of ATPC hydrolyzed per minute and per milligram protein, were expressed in percentage compared to control cells.

Total PLA_2_ specific activity was 20.1 ± 1.8 pmol/min/mg protein in non infected cells, 32.2 ± 2.3 pmol/min/mg protein after short-term *E*. *coli* infection and 42.3 ± 3.5 pmol/min/mg protein after long-term *E*. *coli* infection in absence of inhibitors. PLA_2_ activity of non-infected INS-1E cells (white bars) decreased almost 2.0 and 1.6 fold in presence of EDTA and BEL, respectively, compared to control cells in absence of inhibitors. As expected, the dual (cPLA_2_ and iPLA_2_) phospholipase blocker AACOCF3 almost totally reduced the specific activity to a very low level (panel A). PLA_2_ activity of infected INS-1E cells (black bars) was significantly activated (almost 1.4 fold) compared to non-infected cells. The incubation of INS-1E cells with *E*. *coli* in presence of EDTA caused a significant decrease of PLA_2_ activity, 2.1 fold in comparison with infected cells; BEL decreased PLA_2_ activity 1.2 fold, highlighting that, following *E*. *coli* acute infection, cPLA_2_ activity is mainly responsible for the AA production. Moreover, AACOCF3 almost completely reduced PLA_2_ activity of infected INS-1E cells.

After long-term infection, PLA_2_ activity of INS-1E cells (black bars) was significantly activated (almost 2.1 fold) compared to non-infected cells. The incubation of INS-1E cells with *E*. *coli* in presence of EDTA caused a significant decrease of PLA_2_ activity, 1.5 fold, in comparison to appropriate control; BEL decreased PLA_2_ activity, 2.5 fold, highlighting that, following long-term *E*. *coli* infection, iPLA_2_ activity is mainly responsible for AA production. AACOCF3 almost completely reduced PLA_2_ activity of long-term infected INS-1E cells. Moreover, sPLA_2_ activity was assayed in INS-1E cells after bacterial infection (panel B). No differences in sPLA_2_ activity were found after short- or long-term infection, indicating that sPLA_2_ is not involved in the response of the cells after *E*. *coli* infection.

Bacterial PLA_2_ was also assayed, but its contribution in the bacterial concentration used in our experiments was undetectable.

### cPLA_2_, p-cPLA_2,_ iPLA_2_, COX-1 and COX-2 expressions after *E*. *coli* infection

**Fig 5:**
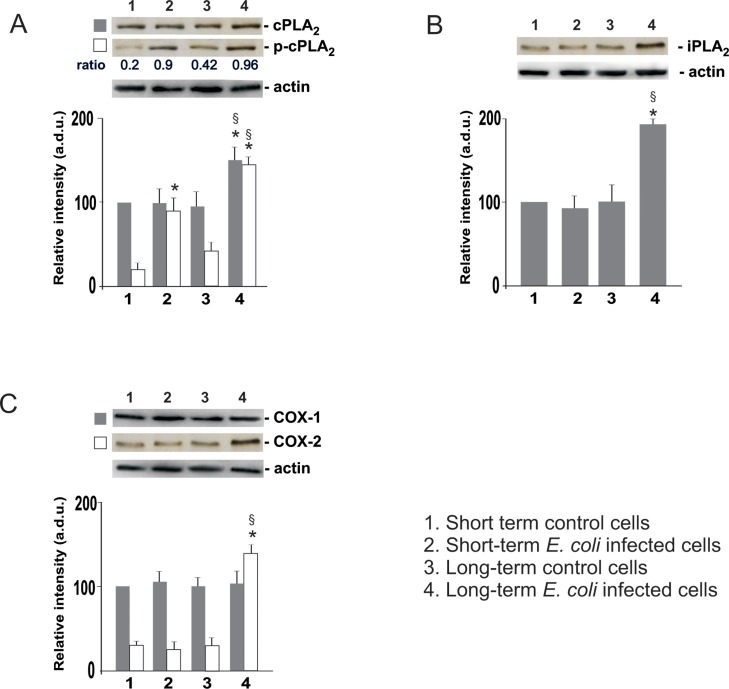
Western blot analysis of cPLA_2_ and p-cPLA_2_ (A), iPLA_2_ (B), and COX-1/2 (C) in INS-1E cells after short and long-term infection with *E*. *coli*. The values, expressed as arbitrary densitometric units (a.d.u.) were obtained by reading the blots using the ImageJ program and are the mean ± SD from three independent experiments (n = 3) performed in triplicate. Control cells: non infected cells. Statistically significant differences, determined by one-way ANOVA and the Tukey *post test*, are indicated (p< 0.05). (*) infected *vs* non-infected cells at the same incubation period; (§) long-term infected *vs* short-term infected cells (line 4 *vs* line 2).

Western blot analyses in Fig 5, panel A, show the total cPLA_2_ and its phosphorylated levels, in INS-1E cells non-infected or infected (acute and chronic infection) with *E*. *coli*.

After short-term infection, no changes in the protein levels of total cPLA_2_ in infected INS-1E in comparison to non-infected cells were observed whereas cPLA_2_ expression increased about 1.5 fold in *E*. *coli* treated cells after long-term infection in comparison to non-infected cells in the same incubation period and to short-term infected cells. The phosphorylated form of cPLA_2_ increased in the cells after short-term infection 4.5 fold (0.9 *vs* 0.2 ratio p-cPLA_2_/cPLA_2_) and after long-term infection 3.1 fold (0.96 *vs* 0.42 ratio p-cPLA_2_/cPLA_2_), in comparison to the respective non-infected cells in the same incubation period. Moreover, long-term infection increased p-cPLA_2_ expression 1.6 fold in comparison to short-term infected cells (line 4 *vs* line 2).

Calcium-independent PLA_2_ expression (panel B) did not change after short-term infection, whereas iPLA_2_ expression increased 1.8 fold in *E*. *coli* treated INS-1E cells after long-term infection in comparison to control cells in the same incubation period (line 4 *vs* line 3) and to short-term infected cells (line 4 *vs* line 2).

Furthermore, COX-2 expression (panel C) significantly increased in the cells after long-term infection 4.6 fold in comparison to the respective control non-infected cells in the same incubation period, and 5.1 fold in comparison to short-term infected cells. No changes in COX-1 expression were observed.

### Transfection of cPLA_2_- and iPLA_2_-siRNA

**Fig 6:**
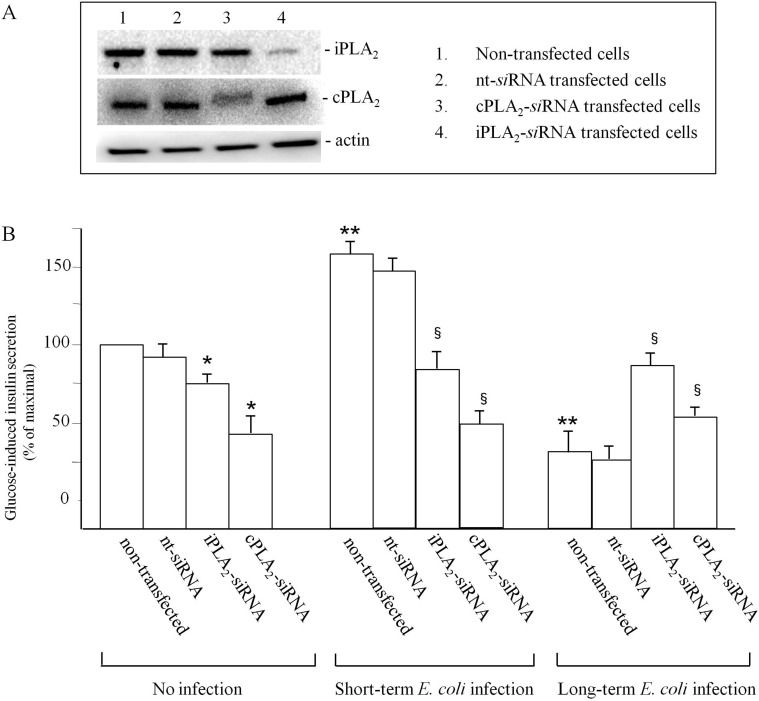
Insulin release in INS-1E cells transfected with PLA_2_-*si*RNAs. (A): cell lysates were immunoblotted to confirm the reduction of PLA_2_ protein levels. (B): insulin secretion of PLA_2_-*si*RNA transfected cells, infected or non infected with *E*. *coli*. Data is expressed as percentage of maximal secretion shown in non-infected/non-transfected INS-1E cells (mean ± SD measured by three independent experiments performed in triplicate), which for glucose stimulation is obtained at 16.6 mM glucose concentration. Statistically significant differences, by one-way ANOVA and the Tukey *post-test* (p< 0.05) are indicated: (*) cPLA_2_- and iPLA_2_-*si*RNA transfected/non infected cells *vs* non-transfected/non-infected cells. (**) non-transfected/infected cells *vs* non-transfected/non-infected cells (equal to 100%) at 16.6 mM glucose concentration w/o transfection. (§) cPLA_2_- and iPLA_2_-*si*RNA transfected/infected cells *vs* non-transfected/infected cells.

In order to shed light on the role played by cPLA_2_ and iPLA_2_ in insulin secretion after short-term or long-term infection, their respective mRNA were silenced using specific *si*RNA. Western blot of INS-1E lysates from three separate preparations of cells after transfection revealed the specificity of *si*RNAs used (panel A): both PLA_2_ protein basal expressions were strongly attenuated in transfected/non infected cells (Fig 6A, lanes 3 and 4, respectively), compared to non-transfected cells (control INS-1E, lane 1) or transfected with non targeting *si*RNA (lane 2). Insulin release was determined in transfected INS-1E cells without *E*. *coli* infection, after short-term infection and after long-term infection and the comparison among the three conditions at the most significant glucose concentration of 16.6 mM, is reported in Fig 6, panel B. In non-infected cells, the insulin release at 16.6 mM glucose concentration significantly decreased 3.3 and 1.5 fold in cPLA_2_- and iPLA_2_-*si*RNA transfected cells, respectively, in comparison to non-transfected INS-1E cells. After short-term infection, insulin release at 16.6 mM glucose concentration in non-transfected/infected cells significantly increased 1.6 fold in comparison to non-transfected/non-infected cells at the same glucose concentration. Moreover, insulin release at 16.6 mM glucose concentration significantly decreased 1.8 and 1.2 fold in cPLA_2_- and iPLA_2_-*si*RNA transfected cells, respectively, in comparison to non-transfected INS-1E cells. After long-term infection, insulin release at 16.6 mM in non-transfected/infected cells decreased 2.8 fold in comparison to non-transfected/non-infected cells at the same glucose concentration (value equal to 100). In these conditions, insulin release by cPLA_2_- and iPLA_2_-*si*RNA transfected INS-1E cells at 16.6 mM glucose concentrations increased 1.7 and 2.5 fold respectively, in comparison to non-transfected/infected cells.

These results demonstrate the involvement of cPLA_2_ and iPLA_2_ activities in insulin release after *E*. *coli* infection and in particular the different responses of the transfected cells to acute and chronic infection. In acute infection, the silencing of iPLA_2_ and, even more so, cPLA_2_ reduces insulin secretion, confirming the involvement of these enzymes. However, in chronic infection, insulin release decreases in comparison to non-infected cells and the silencing of the cPLA_2_ and, even more so, iPLA_2_ determines a rise in the values of insulin release. The results demonstrate that iPLA_2_ is the main factor responsible for the decrease of insulin secretion after chronic infection: its activation leads to the release of the AA metabolism products into the cells, such as prostaglandins, whose effect could be manifested by the reduction of insulin release.

### PGE_2_ production after *E*. *coli* infection

PGE_2_ production was measured in non-infected or short- and long-term infected INS-1E cells. As shown in Table 1, a 1.1-fold increase after *E*. *coli* short-term infection was observed in comparison to the respective non-infected cells. *E*. *coli* incubation in presence of 50 μM AACOCF3 or 2.5 μM BEL decreased PGE_2_ production 1.4 and 1.3 fold, respectively. After long-term infection, PGE_2_ production increased 3.2 fold in comparison to the respective control and the presence of AACOCF3 or BEL reduced PGE_2_ levels 3.5 and 2.8 fold, respectively. The results in presence of BEL demonstrated the cPLA_2_ contribution in PGE_2_ release. In long-term infection experiments, most of PGE_2_ produced could be a result of iPLA_2_ activity.

**Table 1:** PGE_2_ production in INS-1E cells stimulated and non-stimulated by *E*. *coli*.

	Control cells PGE_2_ secretion (pg/ml)	Cells + *E*. *coli* PGE_2_ secretion (pg/ml)
**Short-term infection**		
INS-1E	115 ± 13.8	130 ± 12.1*
INS-1E + AACOCF3	98 ± 8.3^a^	91 ± 7.9^a^
INS-1E + BEL	104 ± 9.9	95 ± 8.9^a^
**Long-term infection**		
INS-1E	122 ± 10.6^b^	388 ± 22.3^b^*
INS-1E + AACOCF3	97 ± 8.5^a^	110 ± 10.1^a^
INS-1E + BEL	108 ± 9.5^a^	138 ± 12.6^a^

**a.** The statistically significant differences in PGE2 production of cultures incubated with PLA_2_ inhibitors in comparison with the respective in absence of inhibitors.

**b.** The statistically significant differences, between long-time infected and non-infected cultures versus the respective short-time infected cultures.

INS-1E cells (8 x 10^5^ cells/well) were pre-incubated for 60 min in culture medium supplemented or not with either 50 mM AACOCF3 or 2.5 mM BEL. The cells were then re-fed with fresh culture medium containing the inhibitors in presence or in absence of *E*. *coli* (10^7^ CFU/well) for 8h (short-term infection) or for 8h and subsequently further incubated for 72h (long-term infection).

Cell culture supernatants were assayed for PGE2 production. Values (means ± SEM) are from three independent experiments (*n* = 3). ANOVA and the Tukey post-test were used to compare PGE2 production in the different experimental conditions (*P* < 0.05). Stimulated cells versus control cultures (not stimulated by bacteria), are indicated by asterisk (*).

### PGE_2_ imbalance causes dysfunction of insulin secretion

**Fig 7:**
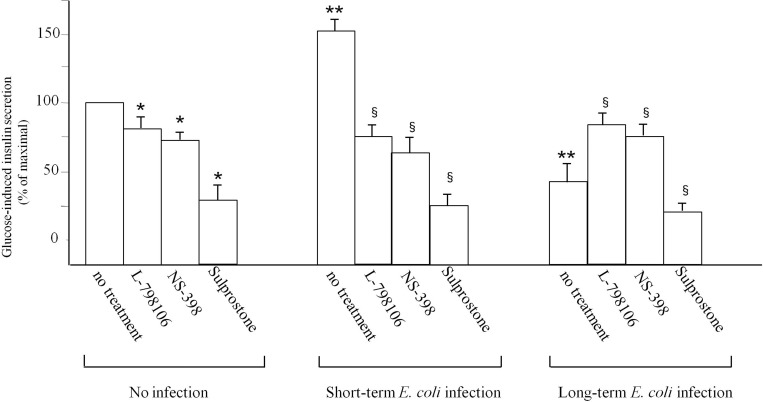
Insulin release in non-infected INS-1E cells, or infected with *E*. *coli* for short-term or long-term. INS-1E were pre-incubated for 60 min in culture medium supplemented or not with 5 μM NS-398, COX-2 specific inhibitor, or 20 μM L-798106, specific EP3 antagonist, or 10 nM sulprostone, specific EP3 agonist. Data is expressed as a percentage of maximal secretion shown in INS-1E cells (mean ± SD measured by three independent experiments performed in triplicate), which for glucose stimulation is obtained at 16.6 mM. Statistically significant differences, by one-way ANOVA and the Tukey *post-test* (p< 0.05) are indicated: (*) Treated non-infected cells *vs* non infected cells with no treatment. (**) Infected cells *vs* non-infected cells (equal to 100%) at 16.6 mM glucose concentration. (§) Treated infected cells *vs* infected cells with no treatment.

The increased production of PGE_2_ raised the possibility of an autocrine-paracrine loop in INS-1E cells after *E*. *coli* infection, causing insulin secretion reduction. For this reason, we hypothesized that it would be possible to modulate insulin secretion by acting on the EP3 receptor. Insulin secretion in non-infected INS-1E cells either after short-term infection or after long-term infection in absence or presence of L-798106, a specific EP3 antagonist, or NS-398, COX-2 inhibitor, or sulprostone, a specific EP3 agonist, was determined. The comparison among the three conditions at the most significant glucose concentration of 16.6 mM, is reported in Fig 7. In non-infected cells the insulin release significantly decreased by 20%, 26% and 73% in presence of L-798106, NS-398 and sulprostone, respectively, in comparison to control INS-1E cells in absence of inhibitors. After short-term infection, insulin release in infected cells significantly increased 1.6 fold in comparison to non-infected cells at the same glucose concentration. Moreover, insulin release significantly decreased by 51%, 58% and 84% in presence of L-798106, NS-398 and sulprostone, respectively, in comparison to short-term infected cells in absence of inhibitors. After long-term infection, insulin release in infected cells significantly decreased by 60% in comparison to non-infected cells. The presence of sulprostone further reduced insulin secretion by 80% in comparison to infected cells in absence of the inhibitor. However, the presence of NS-398 or L-798106 led to a recovery of insulin secretion by 35% and 50%, respectively. The results demonstrate that PGE_2_ production is responsible for insulin release dysfunction after *E*. *coli* infection.

## Discussion

The onset of T1DM in most cases is a gradual process, with a long period of subclinical disease that precedes the onset of clinical disease. For this reason, the identification of the factors responsible for the initiation of the disease process, years before the diagnosis of diabetes, is a goal of great importance in the study of the pathogenesis of T1DM.

Apart from cigarette smoking and alcohol consumption, the remaining cases of chronic pancreatitis are considered idiopathic in nature [43].

Experimental studies on the pathogenesis of pancreatitis have shown that the translocation of bacteria in the mesenteric lymph nodes, peritoneal fluid and blood, and, therefore, in the pancreas, plays a significant role and that the main mechanisms that govern this process are probably related to the proliferation of enteric flora after intestinal dysfunction, damage to the intestinal permeability, and impairment of host immunity [1]. From human cases of pancreatic abscesses, the organisms reported are *Escherichia coli*, *Klebsiella species*, *Proteus species*, *Pseudomonas species*, *Enterobacter species*, *Candida species*, *Staphylococcus aureus*, *Enterococcus fecalis*, *Citrobacter species*, *Bacteroides species*, *Haemophilus influenzae and Mycobacterium tuberculosis* [44]. A certain relationship between diabetes mellitus and *E*. *coli* infection was evidenced, the increased incidence of insulin-dependent diabetes mellitus in *E*. *coli*-infected pediatric patients having been demonstrated [45]. The infection of a host by *E*. *coli* is facilitated by virulence factors, which are coded by virulence-associated genes. *E*. *coli* can express a wide variety of virulence factors, involved in colonization, adhesion and invasion. These factors are adhesins, invasins and toxins which lead to different infection mechanisms [46]. However, EIEC strains are capable of invading and replicating within the cells [28] and therefore of infecting different organs including the pancreas. Despite intensive research, a final conclusion concerning the causal role of microbes in the pathogenesis of T1DM has not been found.

In this study, we used two experimental models, one miming an acute infection (short-term infection) and the other miming a chronic infection (long-term infection) by *E*. *coli* of INS-1E cells, able to secrete insulin in response to elevated glucose concentrations. The glucose concentration-dependence curve for these cells is similar to that of rat islets and, for this reason, INS-1E cells represent a stable and valuable ß-cell model [47].

It has been demonstrated that bacterial infection may reduce or increase the secretion of insulin based on the type of micro-organisms which penetrate the pancreatic tissue [48]. In the experiments here reported, *E*. *coli* was already able to adhere and invade INS-1E cells after 2h of incubation, and, once inside the cell, it could survive within vacuoles. The fate of *E*. *coli* in pancreatic cells is largely unexplored and, at present, its role in affecting PLA_2_ activities and insulin secretion is unknown.

The results obtained in this study show that, after short-term *E*. *coli* infection, PLA_2_ activities were increased and pancreatic cells continue to secrete insulin that results in even higher amounts in the *E*. *coli*-infected cells compared to uninfected ones. When the infection is carried out long-term, insulin secretion is significantly reduced and at the same time extremely high PLA_2_ activities and expressions were observed. We speculated that *E*. *coli* long-term infection determines excessive PLA_2_ activation leading to a significant imbalance in AA concentration. In isolated human islets it has been demonstrated that AA itself, rather than one or more of its metabolites, is responsible for an enhanced secretory response [49]. However, the fact that high concentrations of AA could cause a dangerous imbalance cannot be neglected because this polyunsaturated fatty acid up-regulates COX-2 enzymes which release a high amount of prostaglandins. Our results demonstrated that COX-2 expression is higher after long-term infection in comparison to protein expression in non-infected cells. Instead, short-term infection did not allow new synthesis of COX-2 enzymes. In addition, PGE_2_ production was significantly higher in INS-1E cells after long-term *E*. *coli* infection and the release was significantly reduced in presence of BEL. The residual low production of PGE_2_s in the presence of BEL testifies the significant contribution of iPLA_2_ in PGE_2_ production. Therefore one might speculate that cPLA_2_ plays a leading role in INS-1E under physiological conditions, contributing to the release of insulin. However, after chronic infection, iPLA_2_ was significantly activated, releasing greater amounts of AA and, then, determining COX-2 activation, resulting in increased PGE_2_ production. Consequently, iPLA_2_ could be the isoform primarily responsible for the PGE_2_ imbalance in INS-1E after *E*. *coli* long-term infection. This is confirmed by the results of enzymatic activity shown in Fig 4.

In diabetic islet, it has been demonstrated that increased PGE_2_ production, coupled with increased prostaglandin receptor 3, mediates a negative autocrine-paracrine signalling pathway which antagonizes GLP-1 receptor signalling, *via* a negative effect on cAMP production, contributing to the ß cell dysfunction [50].

In our study, cPLA_2_ and iPLA_2_ differently respond to ßacterial infection after 8h or 72h. After short-term infection, cPLA_2_ activity is higher than iPLA_2_, indicating that cPLA_2_ is the main factor responsible for AA release after acute infection. Differently, after long-term infection, iPLA_2_ was higher than cPLA_2_. These results highlight that the two isoforms play a different role after acute and chronic infection.

The role of cPLA_2_ in the ß-cells is controversial. It seems that cPLA_2_ is not required for the initiation of insulin secretion from ß-cells, but for the maintenance of ß-cell insulin stores [28]; on the other hand, it has been demonstrated that cPLA_2_ß plays an important role in controlling the rate of exocytosis in ß-cells and requires the combined actions of AA and lysophosphatidylcholine [28]. Its overexpression results in severe impairment of insulin secretion through uncoupling of mitochondrial metabolism [29]. Concerning iPLA_2_, it has been demonstrated that it participates in glucose-stimulated insulin secretion in pancreatic ß-cells [24, 25] and a dual role for this enzyme has been proposed, being able to amplify insulin secretion [51] or to contribute to apoptosis [31, 52] when overexpressed.

Numerous studies examining the role of PLA_2_s and AA in insulin release have focused on short-term signalling [53]. However, long term exposure to high levels of fatty acids has been shown to be detrimental to ß-cell function [34, 54] in contrast to the stimulatory short-term effects of exogenous AA and other fatty acids on the ß-cells [53]. It has been demonstrated that acute exposure of pancreatic ß-cells to saturated non-esterified fatty acids, including AA, increases glucose-induced insulin release, whereas chronic exposure results in desensitization and suppression of secretion, followed by induction of apoptosis [55]. We demonstrated that cPLA_2_, releasing AA, is activated by phosphorylation after short- and, even more, after long-term infection. Moreover, after 72h of bacterial incubation, new synthesis of cPLA_2_ and iPLA_2_ proteins indicates a specific response of the cells to counteract bacterial infection.

In view of our findings we propose a model of inflammatory response of INS-1E cells to *E*. *coli* infection in which cPLA_2_ and iPLA_2_, acting in concert, are involved. Their contribution is highlighted by the fact that their silencing by cPLA_2_- and iPLA_2_-*si*RNA transfection significantly reduces the glucose-stimulated insulin secretion in uninfected or short-term infected cells in comparison to non-transfected cells. When, after long-term *E*. *coli* infection, INS-1E no longer respond to high concentrations of glucose, the *si*RNA-iPLA_2_ transfection allows a response to glucose with consequent insulin secretion from INS-1E cells to be restored, even if to a lesser extent compared to that of non-infected cells. In particular, the insulin secretion is higher in *si*RNA-iPLA_2_ than in *si*RNA-cPLA_2_ transfected cells, highlighting that iPLA_2_ plays a main role in insulin secretion after chronic infection of INS-1E cells. Thus, iPLA_2_ could represent a therapeutic target to moderate the AA concentration imbalance, presumed responsible for the insulin secretion reduction in damaged ß cells.

Moreover, insulin release, increased after short-term infection, was significantly reduced in presence of L-798106, PGE_2_ receptor antagonist, NS-398, COX-2 inhibitor, and sulprostone, an EP3 agonist, demonstrating that PGE_2_ are responsible for the INS-1E dysfunction. Instead, insulin secretion, significantly reduced after long-term infection, is restored in presence of NS-398 and L-798106, further confirming the PGE_2_ role after *E*. *coli* infection. We hypothesized that an increase in insulin synthesis after short-term infection could be the initial response to infection of cells with the activation of phospholipases and COX-2, releasing PGE_2_, which, in balanced amounts inside the cell, would have a stimulatory effect on insulin secretion. In fact, it has been demonstrated that the phospholipases participate in amplifying glucose-induced insulin secretion [27, 56].

Conversely, when the infection continues for a longer time, the bacterial growth within the cells would cause excessive phospholipase and COX-2 activation, leading to an imbalance in AA and PGE_2_ concentration which may be responsible for the damage to INS-1E and consequently for the reduction of insulin secretion. In fact, in these conditions, blocking PGE_2_ synthesis or blocking the binding to their receptor, reduces their dangerous effect on INS-1E and restores insulin secretion.

So the main “culprits” as responsible for the reduction of insulin secretion would not be primarily AA and PGE_2_, which are able to induce, at physiological concentrations, an increase of the above synthesis, but the excessive concentration of AA and PGE_2_ within the cells. An imbalance in their concentration would be the mechanism by which *E*. *coli* would lead to the dysfunction of INS-1E. In this regard, it has been demonstrated that sulprostone and PGE_2_, are capable of eliciting a dose-dependent decrease of insulin secretion in glucose-stimulated INS-1 cells [50].

The *E*. *coli* infection is the result of the cumulative effect of numerous molecules acting on different target proteins of intracellular signalling pathways, by changing the functions of the host cell. In particular, type III secretion system (T3SS) and secreted proteins EspA, EspB and EspD, form a traslocom that is essential for protein secretion and for the translocation of multiple effectors into the host cells. These molecules have biochemical functions that make *E*. *coli* able to exert an accurate control of the host cell [57, 58] and are able to trigger cross-talk between bacterial and host cells [59]. It has been shown that molecules produced by *E*. *coli* are able to act on specific signalling pathways that can keep the infected cells alive and consequently may allow the bacteria to multiply within them and to colonize other tissues [60]. In particular, NleE, NleB, NleC, NleD and NleH are targeting specific proteins within inflammatory signalling, which presumably allows the bacteria to establish infection and avoid immediate elimination by the host innate immune response [61]. It has also been demonstrated that Shiga toxin-producing *E*. *coli* induce the activation of intracellular second messenger molecules, including inositol triphosphate and intracellular calcium, in infected eukaryotic cells in tissue culture [62–64]. Moreover, bacteria are able to cause post-translational modifications in the host cells through a variety of bacterial effectors present on their surface or secreted. These effectors can interact with intracellular proteins, by implementing different post-translational modifications. In particular, it has been shown that *E*. *coli* is capable of determining the deamidation of Rho GTPases through the production of CNF-1 (Cytotoxic Necrotizing Factor-1) [65]. Previous studies suggested that the ability of E. coli to raise intracellular calcium levels and generate diacylglycerol (DAG) led to the proposal that EPEC activates calcium- dependent protein kinases, including protein kinase C (PKC), in host epithelia [66]. It has been also reported that activation of PKC results in up-regulation of iPLA_2_ß expression that leads to activation of RhoA/Rho kinase/CPI-17 signalling [67, 68]. In this way, *E*. *coli* could regulate iPLA_2_. It has also been demonstrated that the three mitogen- activated protein kinases (MAPK), ERK1/2, p38, and JNK were phosphorylated in E. coli-infected human colonic cell lines T84 [67] and the tyrosine phosphorylation of host cell proteins [65].

As the infection strategy used by E. coli is post-translational modification, which targets central signalling pathways in the host cell, such as the NF-kB and MAP kinase pathways, it is likely that PLA_2_ is, in turn, activated by MAPK and by increasing intracellular calcium concentration.

We realize the fact that this study was only conducted on INS-1E cells and that further studies are needed to demonstrate the presence of this mechanism also in other models using several types of pancreatic cells. For the time being, the results provide a key to understanding the mechanisms by which *E*. *coli* could damage pancreatic cells.

In conclusion, PLA_2_s, and mainly iPLA_2_, play a key role in the response to the chronic *E*. *coli* infection of INS-1E cells, by producing AA, the COX-2 substrate for PGE_2_ synthesis. Further studies on the ability of the bacteria to modulate insulin secretion are needed to understand the mechanism through which they could cause diabetes, in order to develop strategies for the prevention of insulin imbalance and for the implementation of new therapeutic approaches.
